# Network analysis of headache diagnoses using international classification of headache disorders, 3rd edition

**DOI:** 10.3389/fneur.2025.1526037

**Published:** 2025-01-30

**Authors:** Pengfei Zhang, Thomas Berk

**Affiliations:** ^1^Department of Neurology, Beth Israel Deaconess Medical Center, Harvard Medical School, Boston, MA, United States; ^2^Neura Health, New York, NY, United States; ^3^Thomas Jefferson University Hospital, Philadelphia, PA, United States

**Keywords:** headache classification, network analysis, migraine, centrality measure, disease classification

## Abstract

**Background and objective:**

The International Classification of Headache Disorders, Third Edition (ICHD-3), significantly influences clinicians’ understanding of headache disorders. In this study, we aim to elucidate how the hierarchical structure of ICHD-3 shapes the understanding of interconnectivity among headache disorders.

**Methods:**

A network comprises elements known as “nodes,” with the connections between them referred to as “edges.” In our study, a node represents a headache diagnosis that meets at least one ICHD-3 diagnostic criterion of the ICHD-3. We developed two network models for ICHD-3: a non-hierarchical model, where edges are only formed by cross-references found within the text of diagnoses, and a hierarchical model that incorporates the ICHD-3’s structural organization by adding extra edges between sections and their subsections. We identified the top 10 disorders in terms of their centrality, which assesses their popularity, their role as bridges in the network, and their proximity to other disorders. These measurements are calculated using the network’s degree, betweenness, and closeness centrality.

**Results:**

Both our models contain 387 nodes. The choice between a non-hierarchical or hierarchical model affects which diagnoses occupy the top 10 centrality nodes. In both models, migraine and medication-overuse headaches consistently rank among the top 10 diagnoses according to all three centrality metrics. The hierarchical model includes a greater number of secondary headache diagnoses among its top 10 compared to the non-hierarchical model.

**Conclusion:**

Migraine and medication overuse headaches are the most interconnected nodes in ICHD-3. The addition of a diagnostic hierarchy facilitates the unification of secondary headaches, which would otherwise be considered isolated, miscellaneous diagnoses. When interconnected hierarchically, these secondary headache diagnoses become the majority of the most well-connected nodes in our field.

## Introduction

Network analysis is a widely used technique for studying structures of complex networks. Examples of network analysis in headache research include migraine neuroimaging data and epidemiology studies on migraine comorbidities (1–3).

However, network analysis does not have to be limited to the basic science arena: X (formerly Twitter) and Facebook have undergone extensive network analysis by sociologists (4, 5). In the humanities, projects such as “Six Degrees of Francis Bacon” have also used network analysis to identify influential figures in early modern literary circles (6). Indeed, identifying a set of finite objects and their connections enables network analysis to reveal key ‘hubs,’ where clusters of connections converge, and ‘brokers,’ which serve as bridges between major groups.

Headache disorders can be understood in a complex network, where headache diagnoses are related to each other phenomenologically (e.g., triptan overuse headache and chronic migraine) or as members of the same differential diagnosis (e.g., sex headache and RCVS). They can also relate to each other pathophysiologically or anatomically (e.g., SUNCT and hemicranias continua). *The International Classification of Headache Disorders* (ICHD-3) provides ample discussions in the description and comment sections describing the above connections between headache disorders (7). As such, the text of ICHD-3 may be understood as a curated dataset readily available for network analysis.

The objective of this network analysis study is twofold: to investigate how headaches are connected through the ICHD-3 based on the descriptions of each diagnosis within the criteria. In addition, we also seek to elucidate how the hierarchical nature of ICHD-3 affects how headaches are connected.

## Methods

All data for this study are derived directly from the electronic copy of ICHD-3. This project uses the same dataset as “The Structure and Organizations of ICHD-3 Differential Diagnoses through DiffNet: A Pilot Study,” an article exploring the relationship between differential diagnosis sets in the ICHD-3 (8).

Since this study does not involve patient-related data, ethics board approval was neither required nor obtained.

### Definitions

**Figure 1:**
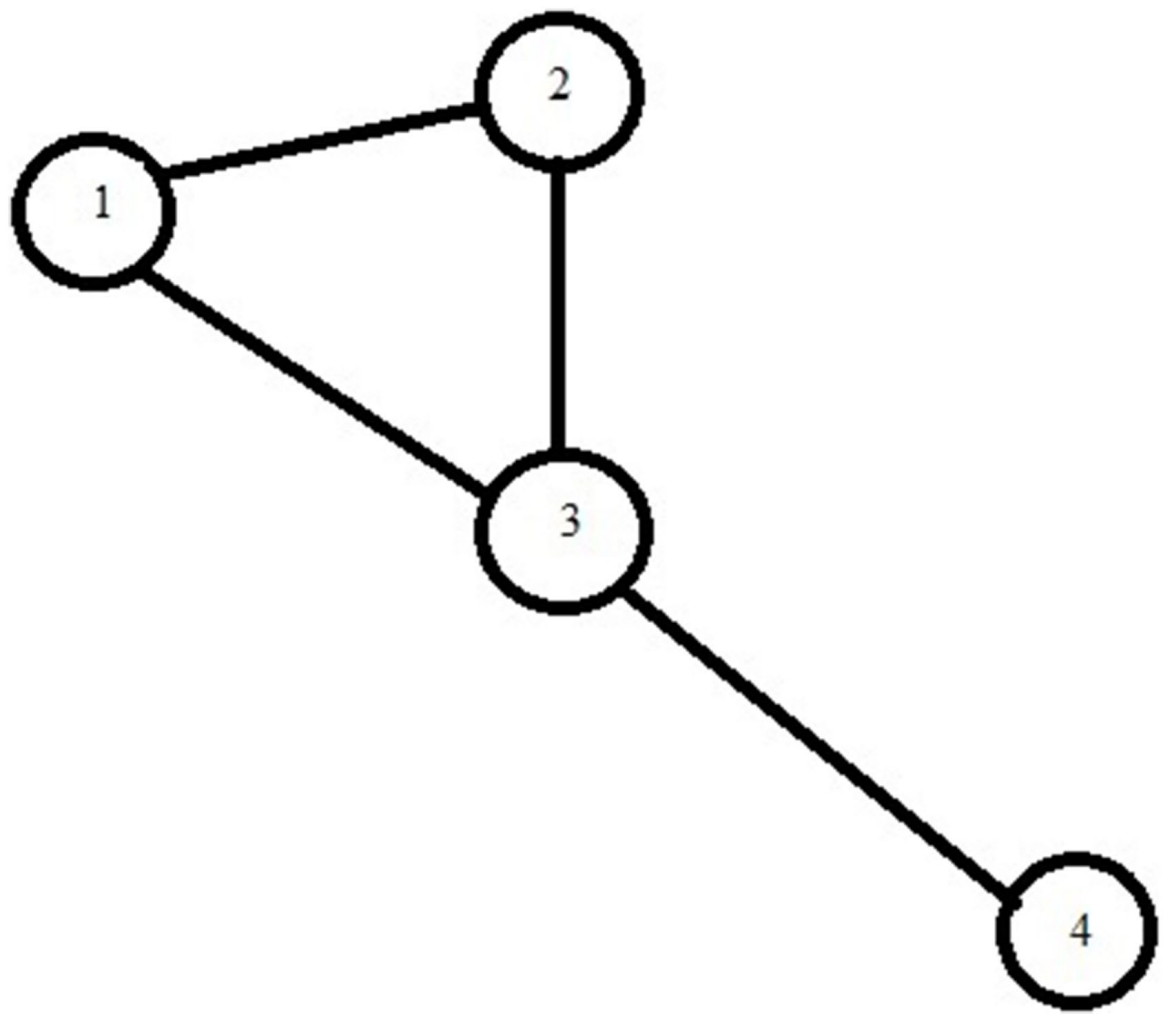
Example of a simple mathematical graph.

A graph G can be defined by two sets—the set of nodes, denoted as V, and the set of edges, denoted as E. For example, consider the graph shown in Figure 1.

We can describe this graph by:

G = (V, E) where.

V = {1, 2, 3, 4}.

E = {(1, 2), (2, 3), (3, 1), (3, 4)}.

*Note that the edge (a, b) is equivalent to edge (b, a). For example, (6, 4) and (4, 6) are equivalent.

Our model defines a node as a headache disorder (i.e., headache diagnosis) identified by at least one ICHD-3 diagnostic code. If a diagnosis is represented by duplicate codes (for example, “migraine” is listed as both 1 and A1), then we count both codes as the same node.

By definition, an edge between two headache disorders exists if one disorder is mentioned explicitly by the other in the content of an ICHD-3 criterion through a diagnostic code (The content of an ICHD-3 diagnosis is defined as the text between the diagnostic code and the following diagnostic code. Bibliographies and tables of contents are excluded).

For example, “primary stabbing headache” (ICHD-3 code 4.7) and “short-lasting unilateral neuralgiform headache attacks” (ICHD-3 code 3.3.1) are both nodes in our graph. An edge also exists between “primary stabbing headache” and “short-lasting unilateral neuralgiform headache attacks” since the latter is mentioned under the content of the former.

Finally, degrees of separation are defined as the number of edge connections from a given node.

### The models

To investigate how the hierarchical nature of the ICHD-3 alters network structure, we developed two models for the ICHD-3: a non-hierarchical model and a hierarchical model. In the non-hierarchical model, only cross-references in the subsections qualified as edges, as detailed above. In the hierarchical model, the structure of the ICHD-3 is taken into account by establishing additional edges between sections and their subsections. For example, an edge exists between 11.5 and 11.5.2. An edge also exists between 11 and 11.5.2. However, 11.4 is not automatically connected to 11.5.

Our model only includes cross-references that are explicitly cited in the text through an ICHD-3 code. Cross-references that are mentioned but not cited by an ICHD-3 code are not included. Our decision to exclude these “missing” cross-references arises from two concerns: (1) Inclusion of non-ICHD-3 would imply expanding the network beyond ICHD-3 diagnoses and the scope of our inquiry. (2) The inclusion of ICHD-3 diagnoses that are mentioned but not coded would introduce imprecision in our model. For example, ischemic stroke is explicitly mentioned by comments on migraineous infarction, ICHD-3 code 1.4.3. However, we did not include “ischemic stroke” as a node in our model since it is not an ICHD-3 diagnosis per se. In hypnic headaches (4.9), medication overuse headaches are explicitly mentioned, but neither is cross-referenced as an ICHD-3 diagnosis. As a result, neither is included as an edge. Our rationale is that the inclusion of one or more of those diagnoses here would introduce imprecision in our methodology—i.e., we would have to arbitrarily decide, apart from ICHD-3’s internal rationale, which of the medication overuse headache encodings (i.e., which of the 8.1 subsections) should be related to hypnic headache. We will discuss the implication of this exclusion further in the strengths and weaknesses section.

### Centrality calculations

Centrality is a way of identifying important nodes in a network. In network theory, three commonly used methods of calculating centralities – called centrality measures – are degree, betweenness, and closeness centralities (5, 9). We obtain the top 10 most important nodes for each model by calculating each of these centrality measures. These measurements are defined below. We used definitions in accordance with those within our instrumentation software library, NetworkX, to guarantee consistency:

Degree centrality accounts for the most popular nodes in our network by counting the number of first-degree neighbors (10). Betweenness centrality measures the frequency at which a node occupies a position in the shortest path between any two nodes in the graph. These nodes may not necessarily be well connected by degree but serve as important bridges between major groups within a graph. Formally, the betweenness-centrality for a node v, $C_{B}\left(v\right)$, is defined as


$$C_{B}\left(v\right)=\sum_{s,t\in V}\frac{\sigma \left(s,t|v\right)}{\sigma \left(s,t\right)}\text{,}$$

where $\sigma \left(s,t\right)$ is the shortest path between node s and node t is the number of paths passing through node v. If s=t, then the shortest path is defined as 1. If v is either s∨t, then σ(s,t|v is defined as 0 (10). Finally, closeness centrality measures the shortest distance between a node and all other nodes in the graph. This measures how connected a node is to the rest of the network. Formally, the closeness centrality for a node u is defined as


$$C\left(u\right)=\frac{n-1}{\sum_{v=1}^{n-1}d\left(v,u\right)}$$

where d is the shortest path distance (using Dijkstra’s algorithm) between *v* and *u*; *n* is the number of nodes in the graph (10).

Each centrality measure used in our analysis has distinct clinical and pathophysiological implications: Disorders with a high degree of centrality have numerous direct connections, indicating a broader range of differential diagnoses clinically or a sharing of pathophysiological features with many other disorders. Disorders with high betweenness centrality act as bridges between different clusters or hubs within the network.

If we consider “edges” in our model as elements of differential diagnosis, these disorders straddle various categories, influencing both the clinical approach and the diagnostic workup. Pathophysiologically, they connect disparate categories of disorders. Finally, disorders with high closeness centrality are “closer” and thus more “similar” to all other headache disorders, whether in terms of pathophysiology or clinical manifestation. Although less directly clinically relevant, these disorders could potentially serve as models for understanding headache disorders more broadly.

### Instrumentations

Both the list of nodes and the list of edges are produced through a text version of ICHD-3. Minor formatting problems are adjusted manually. The list of nodes is simply the set of all ICHD-3 diagnosis codes. A list of all edges is generated algorithmically using the above definition through custom backend software through Haskell, a general-purpose programming language. Both lists were manually verified and corrected for quality and completion.

We conducted our network analysis using Networkx version 2.5 and Python Louvain, which are network analysis libraries used within the Python programming environment. Portions of the source code for analyzing network characteristic were adapted from previous studies by Ladd et al. and from Networkx documentations, with appropriate permission (9, 10). The resulting figures were exported in GEXF format and visualized using Gephi, an open-sourced network analysis and data visualization software.

## Results

There are 387 nodes in both of our models. The non-hierarchical model has 716 edges with an average degree of separation of 3.70. The hierarchical model has 1,368 edges with an average degree of 7.07.

**Figure 2:**
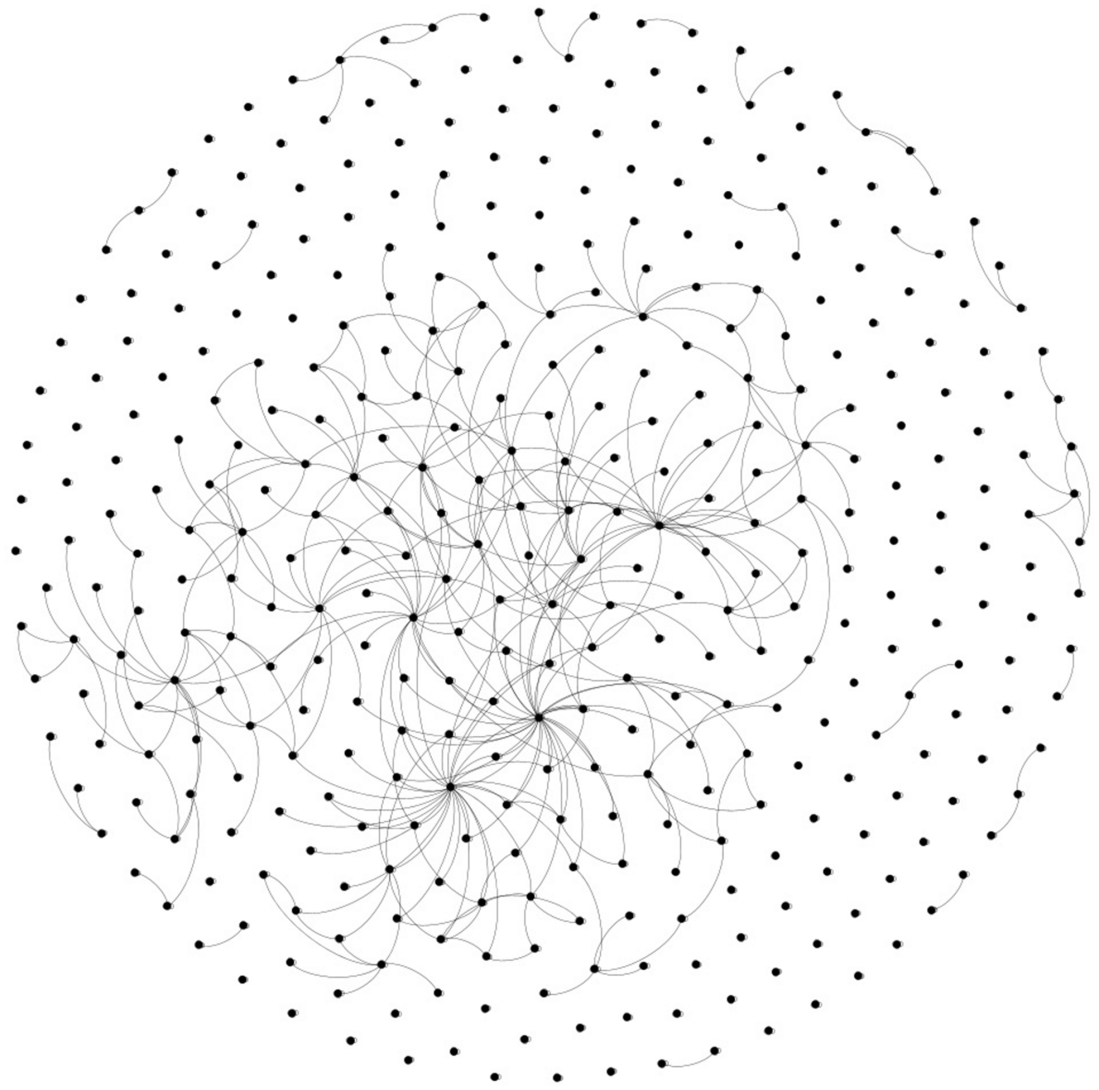
Non-hierarchical model.

**Figure 3:**
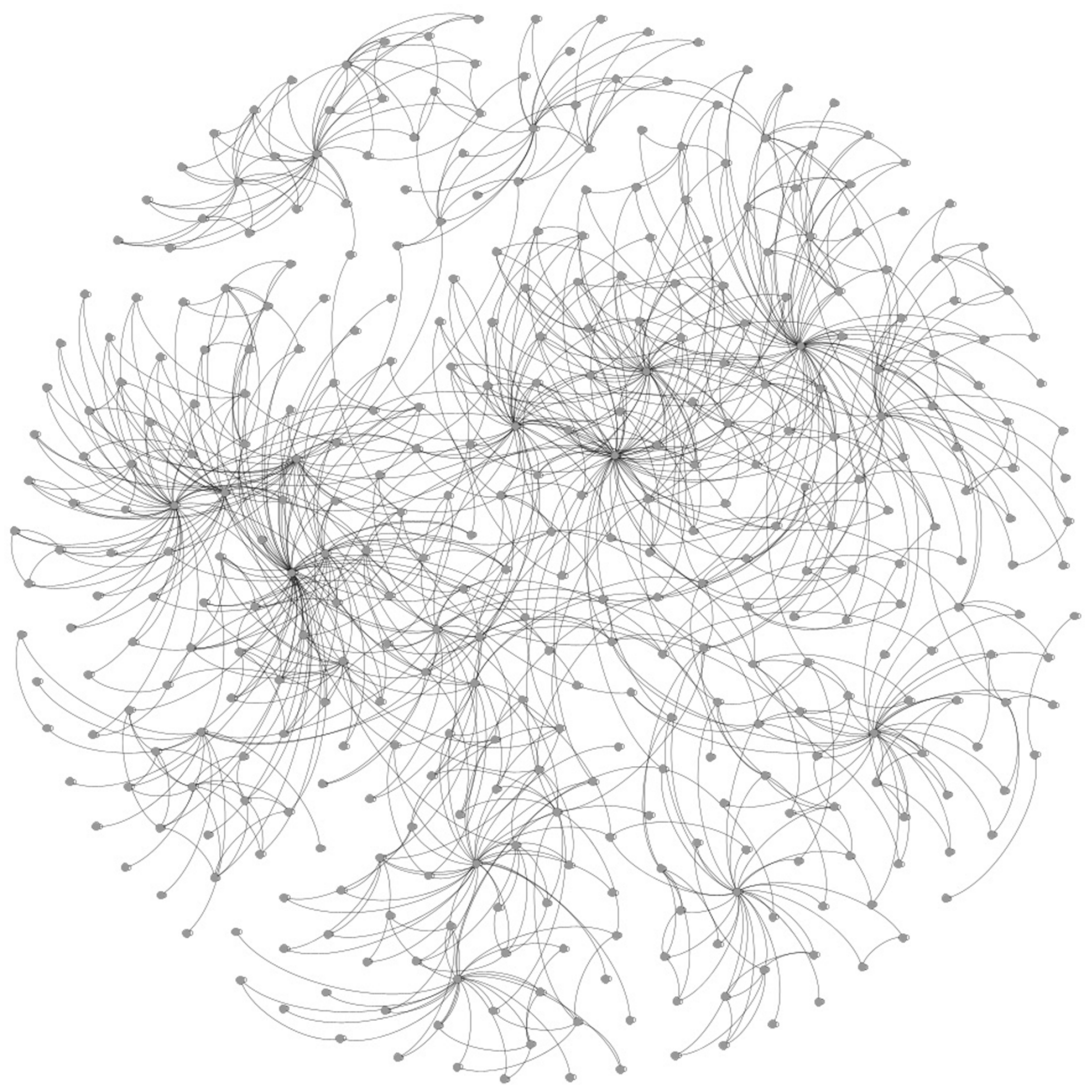
Hierarchy model.

**Figure 4:**
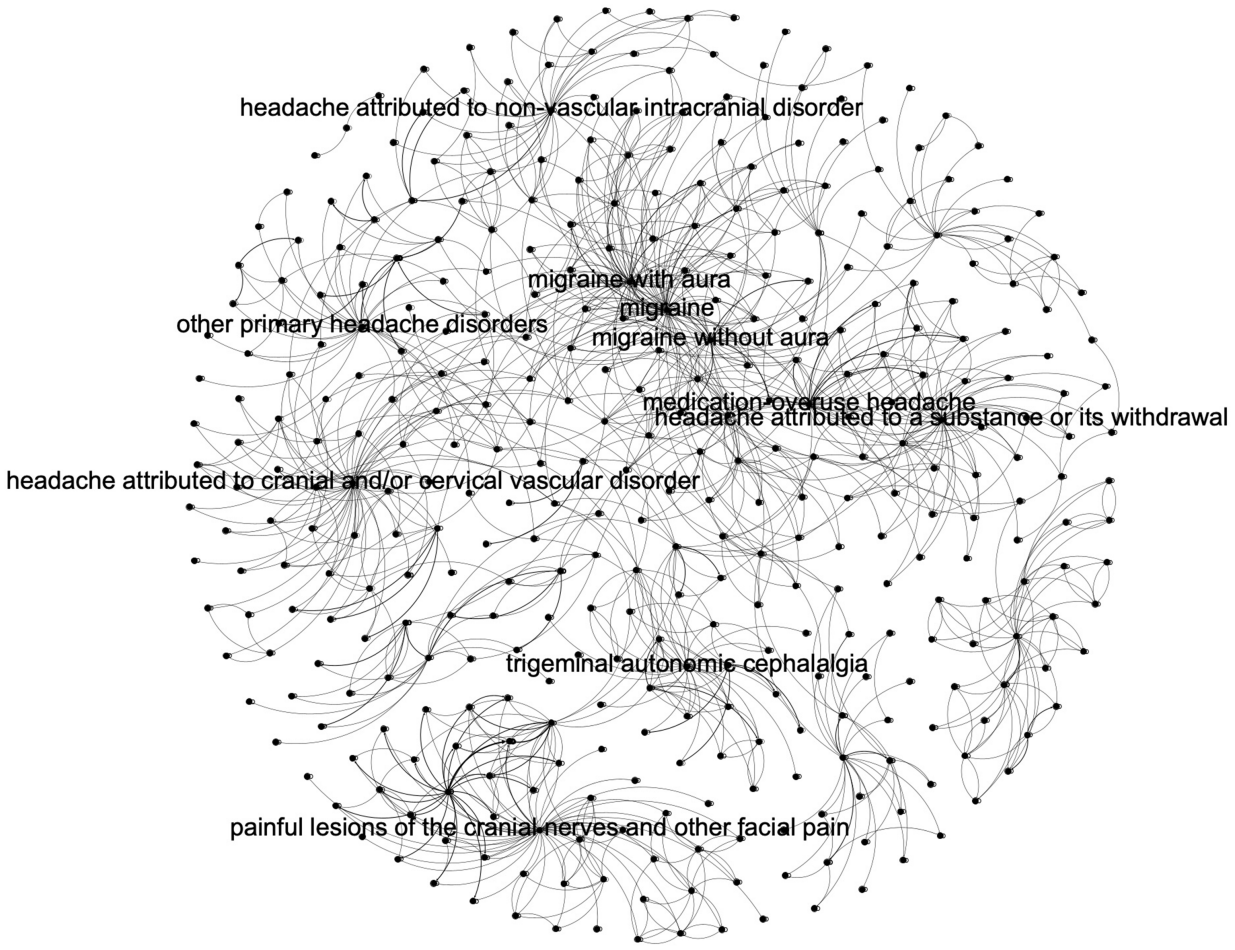
Hierarchy model with top centralities labeled.

Our non-hierarchical model is shown in Figure 2. The hierarchical model is shown in Figure 3. A labeled version of the hierarchical model is shown in Figure 4 with degree centralities labeled. We calculated centrality measures with three different methods: degree centrality, betweenness centrality, and closeness centrality. The top 10 disorders from each of the centrality measures are shown below.

### Degree centrality

Non-hierarchical model top 10 by degree centrality:

1. Migraine without aura
2. Migraine with aura
3. Medication-overuse headache
4. Migraine
5. Chronic tension-type headache
6. Cluster headache
7. Trigeminal neuralgia
8. Chronic migraine
9. Frequent episodic tension-type headache
10. Cervicogenic headache

Hierarchy model top 10 by degree centrality:

1. Migraine
2. Headache attributed to cranial and/or cervical vascular disorder
3. Painful lesions of the cranial nerves and other facial pain
4. Migraine with aura
5. Headache attributed to a substance or its withdrawal
6. Migraine without aura
7. Headache attributed to non-vascular intracranial disorder
8. Trigeminal autonomic cephalalgia
9. Other primary headache disorders
10. Medication-overuse headache

For degree centrality measurements, the top three classes of headache disorders - migraine, tension-type headaches, and trigeminal autonomic cephalalgia – are expected to occupy the top 10. Primary headaches dominate the top 10 in the non-hierarchy model, whereas secondary headaches are unexpectedly more prevalent in the hierarchy model. The implication is, therefore, that a hierarchical model allows secondary headaches to become more apparent to users of the ICHD-3.

### Betweenness centrality

The top 10 betweenness centrality nodes for the non-hierarchy model are:

1. Medication-overuse headache
2. Migraine without aura
3. Migraine with aura
4. Cluster headache
5. Migraine
6. Trigeminal neuralgia
7. New daily persistent headache
8. Headache attributed to cerebral venous thrombosis
9. Hemiplegic migraine
10. Nitric oxide (no) donor-induced headache

The top 10 betweenness centralities for the hierarchy model are as follows:

1. Migraine
2. Cluster headache
3. Trigeminal neuralgia
4. Headache attributed to long-term use of non-headache medication
5. Painful lesions of the cranial nerves and other facial pain
6. Nitric oxide (no) donor-induced headache
7. Headache attributed to cranial and/or cervical vascular disorder
8. Headache attributed to human immunodeficiency virus (HIV) infection
9. Headache attributed to infection
10. Medication-overuse headache

In the non-hierarchy model, primary headaches and their subtypes dominate the top 10 of the betweenness measure and secondary. Headaches continue to be more prevalent in the hierarchy model. However, some of the lesser-known/common headache disorders occurred in both models, including nitric oxide donor-induced headaches, headaches attributed to HIV, and headaches attributed to long-term use of non-headache medications.

This suggests that these less common headache disorders play key roles in connecting different categories of headaches, either in clinical practice (i.e., differential diagnosis) or as bridges between multiple categories of headache disorders pathophysiologically (see discussion).

### Closeness centrality

In the non-hierarchy model, the top 10 closeness centrality are:

1. Migraine without aura
2. Migraine with aura
3. Medication-overuse headache
4. Headache attributed to cerebral venous thrombosis
5. Migraine
6. Chronic tension-type headache
7. Nitric oxide (no) donor-induced headache
8. Chronic migraine
9. New daily persistent headache
10. Cluster headache

In the hierarchy model, the top 30 diagnoses ranked by closeness centrality are as follows:

1. Migraine
2. Nitric oxide (no) donor-induced headache
3. Migraine without aura
4. Headache attributed to the use of or exposure to a substance
5. Cluster headache
6. Acute headache or facial or neck pain attributed to cervical carotid or vertebral artery dissection
7. Headache attributed to cerebral venous thrombosis
8. Tension-type headache
9. Medication-overuse headache
10. Diving headache

Primary headaches continue to dominate the top 10 spaces in centrality measurement in the closeness centrality category. Unexpectedly, nitric oxide donor-induced headache continues to surface in this category. This is, in hindsight, not unexpected since nitric donor headache may be a model for headache disorders in general in migraine/headache research. It is unclear to us why diving headaches occur in the hierarchical model.

## Discussion

Diagnostic criteria are central to headache medicine as the basis of informed clinical decision-making. Since the development of ICHD-3, headache classifications have influenced how practitioners think about and diagnose cephalalgias in both research and clinical settings. This article seeks to reflect on the architecture of the current headache disorder classification system (ICHD-3) using modern network theory.

We present two network models of ICHD-3 and their most important nodes, which are judged by three different measurements of centrality. The occurrence and pattern of each headache disorder in the rankings of centrality measures provide us with insights into the structure and organization of our field as viewed through the ICHD-3.

In both hierarchical and non-hierarchical models, both “migraine” and “medication-overuse headache” stand out among the top 10 diagnoses regardless of the method of centrality measurement. From a network point of view, these two diagnoses serve as major hubs where headache clinical diagnoses converge. Since edges in our model can be interpreted as differential diagnosis considerations, these two disorders are likely to be the most common ones on our differential simply because of how “popular” they are in the network of headache disorders. In other words, they are great diagnostic mimickers. With respect to the former, this should be no surprise to clinicians, as it is famously said that “everything is migraine except when it is not” (15). Our findings also justify the clinical importance of medication overuse as outlined by ICHD-3.

The choice of a non-hierarchical or hierarchical model affects the headache diagnoses that appear as top centrality nodes. In centrality measurement by degree, for example, the non-hierarchical model hubs are dominated by diagnoses that are epidemiologically most common, or at least most commonly seen, in headache clinics and neurological practices: migraine, tension-type headaches, medication overuse headaches, trigeminal neuralgia, and cluster headache (11–14). Once the hierarchical structure of the ICHD-3 is imposed, however, the abundance of various types of secondary headaches, such as cervical vascular and painful neuralgias, push out some of these diagnoses (notably cluster headache, tension-type headache, and trigeminal neuralgia). This intrusion of the secondary headache into the top 10 is also evident when considering betweenness centrality and closeness centrality measurements: secondary headache diagnoses such as substance exposure headaches, vascular disorders, and infections take the top 10 only in the hierarchical model. This is a testament to the power of classification: The addition of headache diagnostic hierarchy allows for the unification of diverse sets of secondary headaches, which would otherwise be considered isolated “islands” of miscellaneous diagnoses. The power of classification is that once connected in a hierarchical fashion, these secondary headache diagnoses form a majority of the most well-connected hubs in our field. This supports the clinical caveat that secondary headaches ought to be at the forefront of all differential diagnostic considerations for headaches; in other words, a proper primary headache diagnosis can be made only by ruling out specific secondary headaches.

The above network observations can be applied clinically: First, our study highlights the utility of not only the criteria but also the hierarchy of ICHD-3 as a framework for clinicians to organize their thinking around secondary headaches. Our study also shows a paradoxical insight regarding migraine and medication overuse headaches. Given their significant centrality within the network, indicating a wealth of differential diagnoses, these conditions are pivotal for mastering differential diagnostic skills. However, they are also notable mimickers of other diseases. Therefore, when encountering patients initially suspected of having these conditions, it is crucial to thoroughly explore alternative diagnoses. Conversely, conditions with low centrality rankings generally have fewer connections to other headache disorders, resulting in more limited differential diagnosis options. Such patients might be “easier” to diagnose due to the limited range of potential differential diagnoses. Finally, our approach of interpreting ICHD-3’s cross-references as differential diagnoses through network theory offers a novel method to further investigate clinical questions related to differential diagnosis.

Our analysis also offers a few unexpected results. For example, nitric oxide donor-induced headache appears in both the top 10 betweenness and closeness centrality but not when centrality is measured by degree. Section 8.1.1 of ICHD-3 provides hints as to why this is the case: ICHD-3 mentions that nitric oxide donor-induced headache induces headache in patients with migraine, tension-type headaches, and cluster headaches. This comment establishes nitric oxide donor headache as a bridge between the top three primary headache disorders. This connection ought not to be disregarded as an idiosyncrasy of an ICHD-3 comment section; rather, it is because of nitric oxide donor-induced headache’s importance in headache pathogenesis that these connections have been made through clinical research to the extent that these diseases are connected in the diagnostic criteria. In other words, nitric oxide donor-induced headache is a bridge because it implicitly connects all three major headache subtypes pathophysiologically.

Trigeminal neuralgia is also important because of the betweenness centrality in both non-hierarchical/hierarchical models. A search of the diagnostic code 13.1.1 through ICHD-3 reveals why this is the case: trigeminal neuralgia serves as a bridge between section 13 and the TAC of section 3. Furthermore, it connects and serves as a unifying point for disorders within section 13, as evidenced by its 10 subsections.

Finally, a few rather “rare” headache disorders serve as connections between major groups of headaches given their appearances in-betweenness centrality: Hemiplegic migraine, for example, earns its rights as a “broker” between headache diagnosis given its connection to vascular headaches and HaNDL. Whereas HIV headache holds a link between 8.1.10 headaches attributed to long-term use of non-headache medication (anti-retroviral) and its clinical similarity to migraine and tension-type headaches (see A9.3).

### Strength and weakness of the study

While the comment section of ICHD-3 aims to provide comprehensive cross-references, these diagnoses are not always identified by an ICHD-3 code. For example, Arnold-Chiari malformation type I and RCVS are both mentioned under Primary Cough Headache, diagnosis criteria 4.1. However, the notes section does not supply a diagnostic code for these two diagnoses. Consequently, our methodology excludes the possibility of establishing edges between these diagnoses. Under both models, RCVS is left out of the differential for primary cough headache. We have not remedied this loophole for methodological rigor. For example, if a headache due to subarachnoid hemorrhage is mentioned without a diagnosis code, it would be an editorial question in deciding whether we are referring to 6.2.2 or 6.2.4.

While our approach of excluding “un-coded” diagnoses allows for precision in our model, we inevitably allow for the potential exclusion of more “hidden” connections in the ICHD-3 network. However, we suspect that the effect of such exclusion is limited since, in our experience with the classification, important differential diagnosis or pathophysiological omission in this fashion is not common. Furthermore, a major function of the comment section of the ICHD-3 is to clarify ambiguity in diagnosis/classification when a headache presentation satisfies multiple primary headache disorders (for example, the precedence of a diagnosis of hemicrania continua over NDPH when the diagnosis satisfies both as discussed in 4.10 notes item 2). We suspect, therefore, that omission of “un-coded” connections among primary headache disorders is less common than omission of secondary headaches (as in the case of Chiari 1 malformation in the example of primary cough headache). This is because direct cross-references are crucial in the guideline to clarify distinctions between primary headaches. Therefore, our model is likely a more accurate portrayal of primary headache connections than secondary headache connections.

Similarly, our model is also limited in that it does not consider diagnoses that do not possess a diagnostic code in the ICHD-3. For example, in criteria 7.6, temporal lobe epilepsy was referenced, but as it is not an ICHD-3 disorder, it is not listed as a node and, therefore, not listed as an edge.

The exclusion of non-headache disorders from our headache classification system is, in our view, inevitable: human diseases generally do not operate within isolated, clearly defined systems, and headache disorders are no exception. The boundary between what is included and excluded in any classification or network analysis model is inherently fluid. Indeed, illnesses are not confined to isolated pathophysiological systems; they also have significant social and cultural dimensions, as exemplified by the Diagnostic and Statistical Manual in Psychiatry. Therefore, striving for a comprehensive model that encapsulates all possible connections impacting headache classification seems unattainable. Essentially, the intrinsic limitation of our model, like any model, is that it presents a compartmentalized view of headache disorders, presupposing that they function within an isolated framework.

Finally, our definition of an “edge” between two disorders does not distinguish whether the connection is phenomenological and/or pathophysiological. This limitation is inherent in disease classification generally; if two disorders share similar pathophysiological pathways, they are likely to exhibit phenomenological similarities and vice versa.

### Implications for future ICHD

Our results suggest some potential implications for future ICHD versions in terms of form and content. Our project demonstrates that it is possible to conduct network analysis of classification guidelines using the ICHD, which allows for precise cross-reference extraction. We, therefore, suggest that future iterations of the classification include comprehensive cross-references of headache disorders and further identify whether each of these cross-references is due to pathophysiological similarities or differential diagnosis considerations. This format will facilitate the ready construction of a comprehensive differential diagnosis for headaches using network techniques (8). It will also allow non-clinician researchers to conduct exclusively network analysis of pathophysiological cross-references in headaches, allowing for the discovery of potential future targets for research.

Given the significant influence of ICHD’s hierarchy demonstrated in our study, we recommend that future versions of the classification include a hierarchical arrangement of “other primary headaches.” This would provide clinicians with a streamlined framework for understanding what may initially appear as disparate disorders, similar to how the existing hierarchy simplifies the conceptualization of secondary headaches.

As to the potential impact on the content of the ICHD, although criteria for headache disorders will inevitably change in future iterations of our classification, the relationships between disease entities – i.e., their cross-references, whether because of clinical phenomenology or pathophysiological relationships – should be relatively stable. For example, that TIA is a differential diagnosis of migraine with aura will remain immutable regardless of the changes in the definition of the latter. In constructing the content of future ICHDs, we recommend that special attention be paid to how a change in criteria does or does not change cross-reference materials.

## Conclusion

Migraine and medication overuse headaches are the most well-connected nodes within the ICHD-3, acting as major hubs where various clinical headache diagnoses converge. In our model, edges represent differential diagnosis considerations, making these two disorders among the most commonly encountered in differential diagnoses due to their high “popularity” within the network of headache disorders. The addition of a diagnostic hierarchy enhances the integration of secondary headaches, which might otherwise be considered isolated, miscellaneous diagnoses. For example, in centrality measurement by degree, the non-hierarchical model’s hubs are dominated by what are considered the core conditions of headache medicine: migraine, tension-type headaches, medication overuse headache, trigeminal neuralgia, and cluster headache. When connected hierarchically, secondary headache diagnoses become some of the most significant diagnoses in our field.

## Data Availability

The raw data supporting the conclusions of this article will be made available by the authors, without undue reservation.
